# Supplementary material for: Plasmodium blood stage development requires the chromatin remodeller Snf2L
Source: Nature. 2025 Feb 19;639(8056):1069–75. doi: 10.1038/s41586-025-08595-x (PMC11946908; doi:10.1038/s41586-025-08595-x)

nature portfolio

https://doi.org/10.1038/s41586-025-08595-x

Supplementary information

Plasmodium blood stage development requires the chromatin remodeller Snf2L

In the format provided by the authors and unedited

Plasmodium blood stage development requires the chromatin remodeler Snf2L

Authors

Maria Theresia Watzlowik[1]	maria-theresia.watzlowik@vkl.uni-regensburg.de
Elisabeth Silberhorn[1]	Elisabeth.Silberhorn@vkl.uni-regensburg.de
Sujaan Das[3]	Sujaan.Das@para.vetmed.uni-muenchen.de
Ritwik Singhal[2]	rbs5758@psu.edu
Kannan Venugopal[6,7]	kannan.venugopal@uzh.ch
Simon Holzinger[1]	simon.holzinger@vkl.uni-regensburg.de
Barbara Stokes[6,7]	Barbara.Stokes@glasgow.ac.uk
Ella Schadt[3]	Ella.Schadt@para.vetmed.uni-muenchen.de
Lauriane Sollelis[6,7]	lauriane.sollelis@uzh.ch
Victoria Ann Bonnell[2]	vab18@psu.edu
Matthew Gow[3]	matthew.gow@para.vetmed.uni-muenchen.de
Andreas Klingl[4]	andreas.klingl@biologie.uni-muenchen.de
Matthias Marti[6,7]	Matthias.Marti@glasgow.ac.uk
Manuel Llinás[2,5]	mul27@psu.edu
Markus Meissner[3,*]	markus.meissner@para.vetmed.uni-muenchen.de
Gernot Längst[1,*]	gernot.laengst@ur.de

[1] Regensburg Center for Biochemistry (RCB), University of Regensburg, 93053 Regensburg, Germany.
[2] Department of Biochemistry and Molecular Biology and Huck Center for Malaria Research, Pennsylvania State University, State College, Pennsylvania, United States of America,
[3] Experimental Parasitology, Department of Veterinary Sciences, Faculty of Veterinary Medicine, Ludwig-Maximilians-University, Munich, Germany
[4] Plant Development, Ludwig-Maximilians-University Munich, Planegg-Martinsried, 82152, Germany
[5] Department of Chemistry, Pennsylvania State University, State College, Pennsylvania, United States of America
[6] Institute of Parasitology, Vetsuisse and Medical faculty, University of Zurich, 8057 Zurich, Switzerland
[7] Institute of Infection and Immunity, College of Medical, Veterinary and Life Sciences, University of Glasgow, Glasgow, United Kingdom
* Corresponding authors: Gernot Längst (gernot.laengst@ur.de) and Markus Meissner (markus.meissner@lmu.de)

Table of Content

- Manuskript_Watzlowik
- Supplementary Table 1
- Supplementary Table 2
- Supplementary Table 3
- Supplementary Table 4
- Supplementary Video 1_noKO
- Supplementary Video 2_iKO
- Extended Data Figures 1 to 10
- Supplementary Figure 1

- Supplementary Note 1
    - Phylogenetic analysis – additional data interpretation 3
    - In vitro characterization of *Pf*Snf2L – additional data interpretation 4
    - MNase-Sequencing – detailed data analysis and interpretation 5
- Supplementary Note 2
    - Biophysical characterization of NH125-*Pf*Snf2L interaction – method section 7
    - Biophysical characterization of NH125-*Pf*Snf2L interactions – additional data interpretation 7
    - Sexual commitment conversion assay – detailed method section and additional data interpretation 8
- Additional References 9
- Supplementary Videos legends 11
- Supplementary Tables legends 11
- Supplementary Figure 1 12

Supplementary Note 1

Phylogenetic analysis – additional data interpretation

The *Plasmodium falciparum* genome encodes ten enzymes that are suggested to be CREs. Due to the relative high divergence to well-characterized proteins in *Homo sapiens* or in model organisms such as *Saccharomyces cerevisiae*, a clear homology assignment is not possible. Previously, the SWI2/SNF2-characteristic ATPase domain was used to group the diverse CREs in subfamilies[1]. To classify the ten plasmodial CREs, a reconstitution of the phylogenetic tree with representative proteins of *H. sapiens, D. melanogaster* and *S. cerevisiae* was performed. Following Flaus et al. (2006), the protein sequences were trimmed to their helicase region based on multiple sequence alignments (not shown, amino acids are listed in Supplementary Table 3), and subsequently used for the phylogenetic analysis including all *Pf*CREs (Extended Data Fig. 1a). Eight of the ten *Pf* enzymes could be assigned to the established subfamilies, with a maximum of one protein in each subgroup, and the Mot1 family as well as the Lsh group being unrepresented. Two enzymes could not be classified: PF3D7_0624600 belongs to an Apicomplexan-specific group and the occurrence of PF3D7_0106000 is limited to Plasmodium species. PF3D7_0624600 was historically called ISWI without a conclusive basis for argumentation. According to our phylogenetic analysis and to a study characterizing this enzyme[3], there is no evolutionary proximity to the ISWI group or any other subfamily. PF3D7_0216000, whose Plasmodium berghei ortholog gSNF2 was recently found to be involved in male gametocyte differentiation, could be identified as Snf2-like CRE.

PF3D7_1104200, previously called *Pf*Snf2L based on the similarity to the human Snf2L enzyme[4], belongs to the ISWI-subfamily, as the investigated ATPase region clusters to ISWI-representatives. Further phylogenetic analysis with full-length sequences of the ISWI members from various species throughout the tree of life confirms the assignment of *Pf*Snf2L to the ISWI-subfamily. However, there is no clear homology to one of the ISWI members (Snf2L or Snf2H (*Hs*) / Snf2L or Snf2H (*Mm*) / Isw1 or Isw2 (*Sc*)). This is in accordance with the observation that PF3D7_1104200 is the only ISWI family enzyme in Plasmodium falciparum and potentially evolutionary separated before ISWI-subdivision in higher eukaryotes (Extended Data Fig. 1b).

Multiple sequence alignments within this family and its quantitative comparison unravel regions of conservation and regions of divergence across the protein architecture (Extended Data Fig. 1c, d). Among metazoans, ISWI enzymes show a relatively high degree of conservation across all domains. Comparing Apicomplexan with metazoan enzymes, only parts of the typical ISWI domain architecture are conserved with >50 % pairwise identity for the ATPase region. The auto-regulatory NegC domain is only partially conserved, while the N-terminal region (NTR), which also has an auto-regulatory function, is not identifiable in *Pf*Snf2L. The conservation of the HSS module – usually determining affinity and specificity of ISWI remodeler by recognition of nucleosomes and extra-nucleosomal DNA – is questionable. The SANT domain is relatively conserved, but the existence of the SLIDE domain can only be speculated as the sequence identity is close to random. The HAND domain is not present in *Pf*Snf2L or Apicomplexan orthologues and large insertions are found there. Within Apicomplexans, the sequence identity is moderately enhanced in the (lacking) HAND domain region, suggesting a conserved – but different – domain among this phylum instead.

In vitro characterization of *Pf*Snf2L – additional data interpretation

The DNA/nucleosome binding and remodeling activity of *Pf*Snf2L was characterized with different DNA templates and different sources of purified and recombinant histone octamers (Extended Data Fig. 2). GC-rich and AT-rich DNA molecules of 20 bp length were used to quantify DNA binding affinities of the remodeling enzyme (Extended Data Fig. 2e, f). Competitive EMSA experiments with the FAM-labelled GC-rich DNA and Cy3-labelled AT-rich DNA in the same test tube revealed that *Pf*Snf2L bound preferentially to the AT-rich DNA (Extended Data Fig. 2e). This feature correlates with a specialization of the enzyme to act on the AT-rich genome of *Plasmodium falciparum*. The EMSA data was confirmed by measuring the binding affinity of *Pf*Snf2L towards these DNA molecules by Microscale Thermophoresis [5] (Extended Data Fig. 2f). *Pf*Snf2L shows binding to the short AT-rich DNA with an approximate binding affinity of 7.6 μM whereas no binding to the GC-rich DNA was observed, confirming the EMSA data.

ISWI-type remodeling enzymes bind to the entry/exit site of nucleosomes, interacting with the DNA linker and the nucleosomal core [6,7]. Using two types of nucleosomes in a competitive manner, reconstituted with purified chicken histone octamers on Cy5-labelled NPS-DNA with an additional DNA linker and the Cy3-labelled 147 bp long NPS-DNA, showed the preferential binding of *Pf*Snf2L to the linker DNA containing nucleosome (Extended Data Fig. 2g). *Pf*Snf2L behaves like a typical ISWI-type remodeling enzyme, most probably binding to the nucleosomal DNA entry/exit site.

The nucleosome remodeling activity of *Pf*Snf2L was compared to human *Hs*Snf2L, using nucleosomes reconstituted on long DNA molecules, possessing the 147 bp long NPS sequence and 77 bp long DNA linkers on both sides of the nucleosome (Extended Data Fig. 2h, i). Nucleosome reconstitution was performed with purified calf histone octamers. Equimolar amounts of both remodelers were used for the assay, and the remodeling activity was monitored over time. *Hs*Snf2L exhibited a higher remodeling activity than *Pf*Snf2L, as significant remodeling activity was apparent already after one minute, whereas a similar pattern was observed only after 10 minutes for *Pf*Snf2L. Next, we tested whether the reduced remodeling activity was due to the different amino acid sequence of the calf histone octamers. Recombinant human octamers were reconstituted on the Cy3-labelled DNA template, whereas recombinant Plasmodium octamers were reconstituted on the Cy5-labelled DNA template. Both nucleosomal templates were mixed in equimolar ratio and a competitive remodeling assay with *Pf*Snf2L was performed (Extended Data Fig. 2i, j). Over time the remodeling reaction was similarly efficient with both templates, although the remodeling pattern was different due to the reduced nucleosome positioning capability of the Plasmodium histone octamers [8]. In summary, *Pf*Snf2L remodels both templates with similar efficiency, showing that the enzyme exhibits reduced activity compared to the human *Hs*Snf2L.

Next, nucleosomal templates with increased AT-content within the nucleosome positioning sequence were used instead of the common remodeling templates analyzed in the field. The 207 bp AT-rich sequence exhibited 68% AT-content within the nucleosomal core and the 222 bp template exhibited above 73% AT-content. Both templates were amplified from *Plasmodium falciparum* genomic DNA. Templates with higher AT-content did not give rise to defined nucleosomal complexes (data not shown). Even though the nucleosome assembly with the Plasmodium octamers is very fuzzy, an ATP dependent nucleosome repositioning could be observed with both templates. The highly active human Chd4 remodeling enzyme served as a control. In contrast to the Plasmodium enzyme, however, this enzyme showed no efficient remodeling reaction on these templates when used in its active concentration of 150 nM (Extended Data Fig. 2k, l). *Pf*Snf2L is capable to remodel nucleosomes on AT-rich DNA, and it is apparently specialized to act on AT-rich DNA, as the Chd4 enzyme exhibits almost no remodeling activity on these templates.

MNase-Sequencing – detailed data analysis and interpretation

MNase-sequencing of schizont stage chromatin +/- Snf2L-KO was performed in triplicates, MNase-sequencing of late ring stage chromatin +/- Snf2L-KO in duplicates.
Digestion efficacy of extracted DNA fragments were analyzed with TapeStation 4200 System. Molarity-normalized DNA size distribution was visualized using BioanalyzeR (Extended Data Fig 5a), kinetic is shown as concentration-weighted mean DNA length over time (Fig. 3c). Comparing the size distribution of DNA fragments with the same digestion condition shows no detectable difference in chromatin accessibility between noKO and iKO at schizont stage. However, late ring stage chromatin of iKO parasites is digested to a higher degree than in noKO parasites, indicating a difference in nuclease-accessibility upon *Pf*Snf2L-KO. To ensure the comparability of MNase-Seq data between iKO and noKO, samples with a similar degree of digestion were chosen for sequencing (marked with a dashed line in Extended Data Fig 5a).
For low digested samples, DNA fragments >700 bp were removed using SPRI beads, no size-selection step was applied for high digested samples. 1 ng of high/low digested sample was used as input for NEBNext Ultra II DNA library prep kit (NEB), according to manufacturer's protocol. Libraries were sequenced on the Illumina NextSeq 2000 Sequencing System. Sequenced reads (2x65 bp, paired-end, ~20 Mio reads/sample) were trimmed with skewer 0.2.2 [9] and subsequently mapped to *P. falciparum* 3D7 genome v3.0 (plasmoDB.org, release 52) using Bowtie2 2.3.5 [10] with "--very-sensitive --no-discordant --no-mixed --no-unal" options. Mapped fragments were filtered with deeptools 3.5.0 [11] for a mapping quality of at least 20, a fragment size between 75 -175 bp and proper read pairs. Mapped and filtered fragments were visualized as coverage plots using karyoploteR[12] (exemplary tracks Fig. 2d). While schizont stage nucleosome maps display only minor changes, substantial differences with numerous nucleosomes being changed in positioning, occupancy and fuzziness are found in late ring stage chromatin (Fig. 2d and Extended Data Fig. 5b). The fragments remaining after filtering were used for nucleosome calling with DANPOS (custom version, based on DANPOS3)[13]. Quality control was done with FastQC 0.11.8 [14], qualimap 2.2.2d [14], samtools 1.12 [15] and multiqc 1.9 [16]. The pipeline was implemented with snakemake 5.32.0 [17] and is available at https://github.com/SimHolz/Watzlowik_et_al_2023, MNase-Seq data are available at GEO (GSE228949). Further analysis was done with R.

- DANPOS: Replicate bam files were put into a single directory respectively to be recognized as a single dataset by DANPOS. Nucleosome calling was performed with the following command: python3 danpos.py dpos <Input> -o <outDir> -m 1 -c 51137214 --extend 73 -a 1 -jd 100 -e 1 -z 20 -s 1
  For comparison and differential nucleosome positioning of two datasets, the two inputs are separated by ":" like "iKO_0h_high/:noKO_0h_high/". The differential nucleosome positioning results were adjusted with a custom R script using simulated random nucleosome positions and Benjamini Hochberg Procedure to calculate FDR values and loess-smoothing for improved dyad position calling.
- Positioning changes: A distinction is made between three classes of positioning changes, occupancy changes, fuzziness changes and nucleosome shifts (illustrated schematically in Fig. 2d and defined in Extended Data Fig. 5d). Using differential nucleosomes analysis in late ring stage, affected nucleosomes were identified and the following parameters were defined as changes: Adjusted FDR values (-log10 transformed for occupancy and fuzziness changes, not transformed for shifts) from DANPOS were normalized to maximum change and ranked. Slope=1 was defined as cutoff (Extended Data Fig. 5d). Cutoff values were extracted and applied for differential schizont stage nucleosomes. Among nucleosomes with changed positioning, only the ones within the 20 % best-positioned nucleosomes in both stages were considered (20 % lowest fuzziness-score in noKO: <47

for schizont, <49 for late rings). Positioning changes are indicated (Fig. 2d and Extended Data Fig. 5b), supporting the observation of numerous nucleosomes being remodeled upon *Pf*Snf2L-KO.

- Gene part assignment: Nucleosome positioning changes (<1 % for schizont/~20 % for late rings) were assigned to gene parts obtained from PlasmoDB annotation with a minimum overlap of 99 bp: 5'UTR, protein-coding (= CDS), 3'UTR, intergenic (= no feature assigned), others (= introns, pseudogenes, snoRNA, ncRNA, tRNA, snRNA, rRNA). Clear enrichments of nucleosome positioning changes are found in 5'UTR and intergenic regions (Fig. 2e).
- Profiles: The transcriptional landmark sites (TSS, AUG, STOP, TTS) were extracted from PlasmoDB annotation for each gene on chromosome 1-14, considering only one site per gene. Occupancy profiles were obtained by aligning the coverage 1000 bp upstream and downstream of the extracted landmark for all available replicates and averaging. The corresponding GC-content around landmarks was aligned, averaged, and smoothed accordingly. Comparing iKO and noKO at schizont stage (Extended Data Fig. 5e), the MNase-Seq occupancy profiles overlap largely. However, in late ring stage, the profiles show huge differences with largest differences surrounding the TSS region. In iKO, the region around the start codon (AUG) shows a slight reduction in the occupancy amplitude and a small shift of the nucleosome position directly at the site of the AUG, as shown in the zoom-in of the figure. The nucleosome occupancy around the Stop codon shows a similar but weaker pattern, exhibiting nucleosomes shifted by a few base pairs and occupancy changes. Similar, but weaker effects are observed around the TTS.
- Nucleosome Dynamics Suite: BAM files were used as input for Nucleosome Dynamics program suite [18], utilizing the readBam, nucleR and txstart function. nucleR was run with "--type paired --fragmentLen 175 --thresholdPercentage 50" in addition to default parameters and txstart with "--p1.max.downstream 100" in addition to default parameters.
- +1 Nucleosome Annotation: BAM files of replicates of noKO_24h_high and noKO_0h_high were merged respectively and used as input for Nucleosome Dynamics program suite [18], The 3'-end of the resulting gff from txstart was used as +1 nucleosome dyad annotation. Occupancy profiles around +1 nucleosome were created as described above, resulting in profiles with well-positioned +1 nucleosomes (Extended Data Fig. 5f). The comparison of iKO and noKO shows congruent profiles at schizont stage and highly different profiles at late ring stage. To separate the data according to gene expression, transcripts of all genes at the respective timepoint, normalized as transcripts per millions were subdivided in quantiles. Displaying the +1 nucleosome profiles for the four quantiles separately (Fig. 3d, left panel), shows the most pronounced effect of *Pf*Snf2L-KO on highly expressed genes.
- NFR Width: Distance between +1 and -1 nucleosome as detected by the nucleosome dynamics txstart function was used as NFR width estimation. The distribution of calculated NFR width per gene, was visualized for the four gene expression quantiles. Comparing the four quantiles, highly expressed genes have in total wider NFRs. Comparing iKO and noKO, again shows an increasing (and significant) difference with higher gene expression (Fig. 3d, mid panel). Together, the data suggests a role for *Pf*Snf2L in actively opening the NFRs in context with gene expression activation.
- Mean Fuzziness change calculation: Promoter regions were defined as 1000 bp upstream and 1000 bp downstream of the +1 nucleosomes identified via NucDyn. All called nucleosomes with a minimum overlap of 75 bp with the promoter region were averaged. Mean fuzziness scores were subtracted (iKO-noKO). Only promoters harboring at least 6 called nucleosomes were considered in this analysis. Violin plots of the mean fuzziness change between iKO and noKO show more fuzzy nucleosomes in promoters of genes, that are downregulated upon *Pf*Snf2L. The opposite effect is visible for gene that are upregulated in the absence of *Pf*Snf2L.

Supplementary Note 2

Biophysical characterization of NH125-*Pf*Snf2L interaction – method section

Binding affinities were determined by Microscale Thermophoresis (MST), as described previously [19]. All MST measurements were performed at room temperature using the Monolith NT.015 device (NanoTemper) with standard capillaries. The infrared laser was on for 30 s resulting in a temperature increase of 4 K. Binding reactions took place in a solution containing 100 mM NaCl, 50 mM HEPES, pH 7.6, 0.01% (v/v) Pluronic F-127 and 3 mM $MgCl_2$. A titration series was prepared for each binding analysis with a constant concentration of the fluorescently labeled DNA (20 nM) or protein (50 nM) and increasing concentrations of the inhibitor or DNA, as indicated in the plots. All curves were plotted with the NanoTemper software, and the thermophoresis or initial fluorescence signals were fitted with the K_D equation.
Direct interactions of NH125 (PubChem SID: 329818598), ATP or DNA with the recombinant human, *Plasmodium falciparum* and *Plasmodium vivax* Snf2Lcore enzymes were analyzed by Differential Scanning Fluorometry and Dynamic Light Scattering, using the Prometheus-Panta device (NanoTemper). The melting curve of the proteins (10 μM) was analyzed in a temperature gradient (20-85°C) by recording the absorbance at 330 and 350 nm. The melting temperature is revealed by plotting the first derivative of the absorbance ratio at both wavelengths. The inhibitor NH125 was used at concentrations from 10 to 80 μM, ATP and DNA at 1 mM and 20 μM, respectively. In parallel to the nanoDSF measurement, the particle size distribution was recorded by the Dynamic Light Scattering (DLS) function of the device.

Biophysical characterization of NH125-*Pf*Snf2L interactions – additional data interpretation

After the identification of NH125 by the screening workflow, the binding affinity, binding mode and specificity was determined. Binding affinity was determined by Microscale Thermophoresis, using the His-tag labelled, recombinant *Pf*Snf2Lcore protein with increasing concentrations of NH125. Equilibrium binding was apparent in thermophoresis and initial fluorescence, yielding a K_D of 471 (+/-111) nM for MST and a very similar K_D of 591 (+/-144) nM for the initial fluorescence measurement (Extended Data Fig. 8a). Next, the mode of binding was determined by evaluating the protein stability (nanoDSF) and aggregation behavior (DLS) upon incubation with NH125 (Extended Data Fig. 8b). A protein concentration of 10 μM was used to qualitatively determine the nanoDSF profile of *Pf*Snf2Lcore, revealing two unfolding events with T_{M1} at 27°C and T_{M2} at 49°C. The low temperature transition was reversible, whereas the transition at 49°C resulted in the non-reversible denaturation of the protein. Protein stability was determined for *Pf*Snf2Lcore (10 μM, left panel) in the absence or presence of 10 μM (stochiometric) and 20 μM of NH125. Only a minor destabilization of T_{M2} (-0.5°C) could be observed, suggesting that the overall stability of the domains was not altered due to NH125 binding. However, the simultaneous analysis of particle size by DLS showed an increase of *Pf*Snf2L particle size already at the stoichiometric inhibitor concentration, suggesting conformational changes of the protein that result in aggregation. When analyzing *Pv*Snf2Lcore, changes in protein structure and aggregation were visible only at a concentration of 40 μM, suggesting a much lower binding affinity with a K_D above 20 μM (middle panel). At 40 μM NH125 concentration, PvSnf2L is destabilized (deltaTM 3°C), and the protein also aggregates. In contrast, the human enzyme *Hs*Snf2Lcore (right panel) showed no effects regarding protein stability and aggregation, even at a NH125 concentration of 80 μM. In summary, the results show specific, high-affinity binding of NH125 towards the *Plasmodium falciparum* enzyme.
To address the mode of inhibitor binding, we further analyzed the effect of NH125 towards *Pf*Snf2L in the presence of ATP and DNA (Extended Data Fig. 8c). The left panel shows that DNA (50 μM), ATP (1 mM) and NH125 (10 μM), in contrast to *Pf*Snf2Lcore (10 μM), show only background signals, with no absorption at 330 nM (nano DSF measurement), and no significant DLS signal above 30°C (lowest

panel on the left). *Pf*Snf2Lcore in the presence of NH125 aggregates, as seen before and behaves similarly in the presence of DNA, whereas the interaction of the enzyme with DNA alone results in a smaller complex (middle panel). The nanoDSF data, in turn, suggest that a stable complex between *Pf*Snf2L, NH125 and DNA is formed as the melting curve pattern of the trimeric complex differs from the controls. The stability of the *Pf*Snf2L domains is not affected by the binding of DNA or the inhibitor. The same conclusions can be drawn in the presence of ATP. *Pf*Snf2Lcore did not change its size upon binding of ATP, but ATP changes the nanoDSF melting pattern of T_{M2} (green line), a feature that remained in the presence of ATP and NH125. As the trimeric complex also shows the typical aggregation feature in DLS, it can be concluded that ATP binds to *Pf*Snf2L also in the presence of NH125.
The biophysical data allow us to conclude that NH125 results in a conformational shift and aggregation of the protein (DLS), the inhibitor represses the ATP hydrolysis activity of *Pf*Snf2L (readout of the screen), but it does not bind to the ATP or DNA binding pockets of the protein, as both molecules bind in the presence of NH125 (combined nanoDSF and DLS data).

Sexual commitment conversion assay – detailed method section and additional data interpretation

In order to investigate the effect of NH125 and precisely identify its point of action in gametocytogenesis, we generated a dual reporter line with the aim to quantify sexual commitment and conversion within the same experimental setup. This line was established in the *P. falciparum* strain NF54, wherein the sexual commitment marker AP2-G was endogenously tagged with GFP[20] and the AP2-G target GEXP02 (Gametocyte Exported Protein 02, [21,22]) was endogenously tagged with mScarlet (Extended Data Fig. 10a). To validate the specificity of the gametocyte signals, an AP2-G knockout line was used as negative control for comparison (Extended Data Fig. 10b). The transgenic lines were established using CRISPR/Cas9 genetic engineering. Following transfection, parasites were subjected to Blasticidin S drug selection and cloned by serial dilution. Successful integration into the endogenous locus was confirmed by PCR genotyping (Extended Data Fig. 10c) and clones were phenotyped for commitment and conversion.
Expression of *ap2-g* first peaks in the sexually committed schizont stage and then again in sexual rings, while *gexp02* expression begins in the sexual ring stage and is maintained throughout gametocyte development until stage V (shown in green/red; Extended Data Fig. 10d, e). As an additional readout for gametocytes, we labelled cells with TubulinTracker™ Deep Red (TTDR) (shown in magenta; Extended Data Fig. 10d). TubulinTracker can be used as a stage I to V gametocyte marker, as gametocytes exhibit a polymerized and identifiable tubulin network[23]. To confirm gametocyte-specificity of the TubulinTracker staining, we also determined the signal in asexual parasite stages using the AP2-G KO line as a control (Extended Data Fig. 10e-h). The TubulinTracker staining is highly specific for gametocytes starting at Stage I/Day 2 by flow cytometry, as there is no signal beyond background in the AP2-G KO (Extended Data Fig. 10g). Altogether, these experiments validate the suitability of the three markers to quantify sexual commitment, conversion and gametocyte development. The dual reporter line expressing AP2-GGFP and GEXP02-mSc in combination with TubulinTracker labelling was thus used to comprehensively investigate the effect of NH125 on commitment, conversion, and gametocyte development.

The sexual commitment-conversion assay was optimized from previously published methods [2,24]. Mature schizont-stage parasites were isolated by a 63 % Percoll density gradient centrifugation method and added to naïve RBCs (Day 0). One hour post invasion (hpi), remaining schizonts in the culture were lysed using a 5% sorbitol solution. The resulting highly synchronous parasite cultures were then used to assay the effects of the inhibitor.
At 10 hpi, the commitment-conversion assay was set up in a 96 well plate format, with a starting parasitemia of 0.5% at 2.5% hematocrit in two separate plates with identical conditions. One plate was used for the sexual commitment readout, using the AP2-GGFP signal at the schizont stage. The second plate was used to quantify the parasite multiplication rate (PMR) and the sexual conversion rate in the gametocyte stages. NH125 was added at a range of concentrations, and the plates were incubated under standard parasite culture conditions. 12-14 hours later (22-24 hpi, Day 1) parasites were shifted

to a minimal fatty acid (mFA) medium to induce sexual commitment or a standard control medium (AlbumaxII supplemented with choline chloride) that represses commitment, as previously described[20]. The drug treatment was continued in both growth media. 20-22 hours post induction (42-46 hpi, Day 2), the drug was removed by washing parasites two times in incomplete media. After two washes, cultures were resuspended in complete media. At this stage, the plate reserved for the sexual commitment readout was additionally treated with 50 μM E64 in order to block merozoite egress[25]. Following 6 hours of treatment with E64, the egress-arrested schizonts were stained with Hoechst and subjected to flow cytometry analysis to determine parasitemia and measure the proportion of sexually committed parasites (AP2-GGFP positive). The second 96 well plate was maintained in culture, and the parasitemia was measured 24 hours later (Day 3) to calculate the parasite multiplication rate. At this time point, the culture media was replaced with additional supplementation of Heparin to block the invasion of parasites from this cycle onwards and to eliminate asexual stages[26]. This step is essential in order to accurately measure the sexual conversion rate and count only gametocytes produced during the induction assay. Three days after the addition of heparin (Day 6), gametocytemia was measured using both the GEXP02mScarlet signal and the Tubulin Tracker™ Deep Red signal.
Sexual commitment and conversion rates were calculated based on the flow cytometry data (Fig, 4f, Extended Data Fig. 9e). The sexual commitment rate (measured by AP2-GGFP) was not affected by any of the tested NH125 concentrations (50 nM - 500 nM, panel 1). In contrast, exposure to 200 nM NH125 at 10 hpi led to a significant reduction (~50%) in the production of healthy gametocytes with a polymerized tubulin network (Tubulin Tracker™ Deep Red signal). Consequently, this resulted in a reduced sexual conversion rate, while the PMR was not significantly affected (panel 4). In contrast, AP2-G and GEXP02 signals were not affected by the 200 nM NH125 treatment (panels 2 and 3). Additionally, we tested the addition of NH125 to stage I gametocytes (24 hpi of sexually committed parasites) and found that this did not affect gametocyte development (Extended Data Fig. 9f). Flow cytometry readouts for Tubulin Tracker™ Deep Red and GEXP02 and Giemsa smears across NH125 concentrations are shown in Extended Data Fig. 10i-k.
Combining results across the three markers employed (AP2-G, GEXP02 and tubulin), our data strongly suggest that NH125 acts within early stages of gametocyte development. NH125 did not affect sexual commitment at any of the concentrations tested, but selectively blocked early gametocyte development, i.e., sexual conversion at 200 nM. PMR was also not affected at 200 nM NH125, while higher concentrations affected both conversion rate and PMR. These findings suggest that *Pf*Snf2L acts downstream of AP2-G and its direct targets such as GEXP02 (hence no reduction in the AP2-G and GEXP02 signals), but upstream of the activation of IMC-related genes and before formation of the tubular network (hence a reduction in TubulinTracker™ signal).
Significant reductions in conversion rate but not PMR were also observed in the 3D7, Dd2 and Pf2004 strains (Extended Data Fig. 9g). IC50 values for NH125 showed minor but not statistically significant differences across strains (Extended Data Fig. 9h). Altogether these findings suggest a crucial function of *Pf*Snf2L in early gametocyte development, presumably by contributing to gene expression regulation of stage-specific genes.

Additional References

1. Flaus, A. Identification of multiple distinct Snf2 subfamilies with conserved structural motifs. *Nucleic Acids Research* **34**, 2887–2905 (2006).
2. Brancucci, N. M. B., Goldowitz, I., Buchholz, K., Werling, K. & Marti, M. An assay to probe Plasmodium falciparum growth, transmission stage formation and early gametocyte development. *Nat Protoc* **10**, 1131–1142 (2015).
3. Bryant, J. M. *et al.* Exploring the virulence gene interactome with CRISPR / DC as9 in the human malaria parasite. *Molecular Systems Biology* **16**, (2020).
4. Ji, D. D. & Arnot, D. E. A Plasmodium falciparum homologue of the ATPase subunit of a multi-protein complex involved in chromatin remodelling for transcription. *Mol. Biochem. Parasitol.* **88**, 151–162 (1997).
5. Jerabek-Willemsen, M. *et al.* MicroScale Thermophoresis: Interaction analysis and beyond. *Journal of Molecular Structure* **1077**, 101–113 (2014).
6. Dang, W. & Bartholomew, B. Domain Architecture of the Catalytic Subunit in the ISW2-Nucleosome Complex. *Molecular and Cellular Biology* **27**, 8306–8317 (2007).

7. Längst, G. & Becker, P. B. ISWI Induces Nucleosome Sliding on Nicked DNA. *Molecular Cell* **8**, 1085–1092 (2001).
8. Silberhorn, E. *et al.* Plasmodium falciparum Nucleosomes Exhibit Reduced Stability and Lost Sequence Dependent Nucleosome Positioning. *PLoS Pathog.* **12**, e1006080 (2016).
9. Jiang, H., Lei, R., Ding, S.-W. & Zhu, S. Skewer: A Fast and Accurate Adapter Trimmer for next-Generation Sequencing Paired-End Reads. *BMC bioinformatics* **15**, 182 (2014).
10. Langmead, B. & Salzberg, S. L. Fast Gapped-Read Alignment with Bowtie 2. *Nature Methods* **9**, 357–359 (2012).
11. Ramírez, F. *et al.* deepTools2: A next Generation Web Server for Deep-Sequencing Data Analysis. *Nucleic Acids Research* **44**, W160-165 (2016).
12. Gel, B. & Serra, E. karyoploteR: an R/Bioconductor package to plot customizable genomes displaying arbitrary data. *Bioinformatics* **33**, 3088–3090 (2017).
13. Chen, K. *et al.* DANPOS: Dynamic Analysis of Nucleosome Position and Occupancy by Sequencing. *Genome Research* **23**, 341–351 (2013).
14. Andrews, Simon. FastQC - A Quality Control Tool for High Throughput Sequence Data.
15. Li, H. *et al.* The Sequence Alignment/Map Format and SAMtools. *Bioinformatics (Oxford, England)* **25**, 2078–2079 (2009).
16. Ewels, P., Magnusson, M., Lundin, S. & Käller, M. MultiQC: Summarize Analysis Results for Multiple Tools and Samples in a Single Report. *Bioinformatics (Oxford, England)* **32**, 3047–3048 (2016).
17. Mölder, F. *et al.* Sustainable data analysis with Snakemake. *F1000Res* **10**, 33 (2021).
18. Buitrago, D. *et al.* Nucleosome Dynamics: a new tool for the dynamic analysis of nucleosome positioning. *Nucleic Acids Research* **47**, 9511–9523 (2019).
19. Filarsky, M. *et al.* The extended AT-hook is a novel RNA binding motif. *RNA Biology* **12**, 864–876 (2015).
20. Brancucci, N. M. B. *et al.* Lysophosphatidylcholine Regulates Sexual Stage Differentiation in the Human Malaria Parasite Plasmodium falciparum. *Cell* **171**, 1532-1544.e15 (2017).
21. Warncke, J. D. *et al.* The PHIST protein GEXP02 targets the host cytoskeleton during sexual development of *Plasmodium falciparum*. *Cellular Microbiology* **22**, (2020).
22. Portugaliza, H. P., Llorà-Batlle, O., Rosanas-Urgell, A. & Cortés, A. Reporter lines based on the gexp02 promoter enable early quantification of sexual conversion rates in the malaria parasite Plasmodium falciparum. *Sci Rep* **9**, 14595 (2019).
23. Dearnley, M. *et al.* Reversible host cell remodeling underpins deformability changes in malaria parasite sexual blood stages. *Proc. Natl. Acad. Sci. U.S.A.* **113**, 4800–4805 (2016).
24. Buchholz, K. *et al.* A High-Throughput Screen Targeting Malaria Transmission Stages Opens New Avenues for Drug Development. *The Journal of Infectious Diseases* **203**, 1445–1453 (2011).
25. Salmon, B. L., Oksman, A. & Goldberg, D. E. Malaria parasite exit from the host erythrocyte: A two-step process requiring extraerythrocytic proteolysis. *Proc. Natl. Acad. Sci. U.S.A.* **98**, 271–276 (2001).
26. Boyle, M. J., Richards, J. S., Gilson, P. R., Chai, W. & Beeson, J. G. Interactions with heparin-like molecules during erythrocyte invasion by Plasmodium falciparum merozoites. *Blood* **115**, 4559–4568 (2010).

Legends Supplementary Videos

Supplementary Video 1: normal egress in noKO parasites
Live imaging of one representative egressed noKO schizont. One of n=52 parasites within n=2 biological replicates is shown. Recorded time is indicated.

Supplementary Video 2: impaired egress in iKO parasites
Live imaging of one representative egressed iKO schizont. One of n=96 parasites within n=2 biological replicates is shown. Recorded time is indicated.

Supplementary table 1: Tables displaying the PfSnf2L interactors after Immunoprecipitation and mass spectrometry

Supplementary table 2: Data tables providing the differentially expressed genes after PfSnf2L knockdown.

Supplementary table 3: Data tables showing the genes and domains used for phylogenetic analysis.

Supplementary table 4: File listing the DNA template and Oligo sequences and antibody lists.

Fig1:

Lane 1,2,3 belong to F1b lane 1,2,3

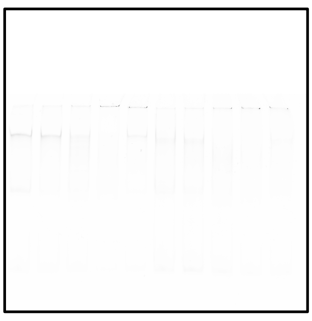

Lane 1,2,3 belong to F1b lane 4,5,6

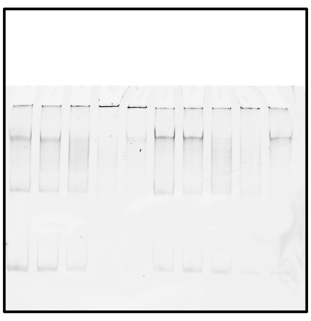

Lane 2,3,4 belong to F1d lane1 ,2,3

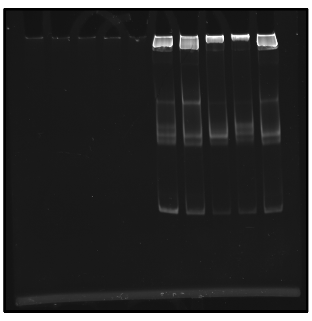

EDF 2:

Lane 1,2,5 belong to EDF2a lane 1,2,3

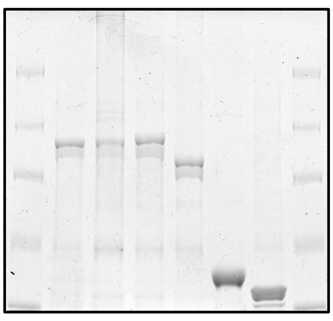

Second last lane belongs to EDF2c lane 1

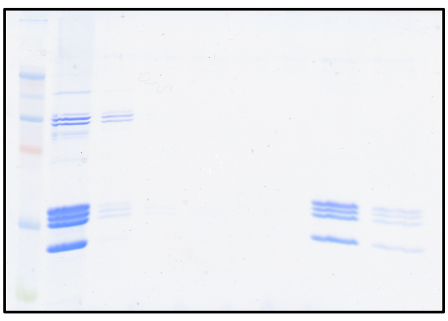

Lane 6,4,7 belong to EDF2c lane 2,3,4

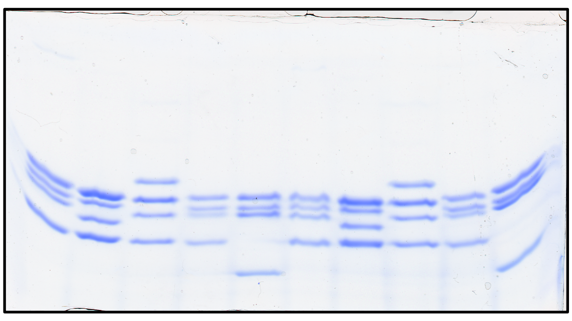

Lane 1 belongs to EDF2d lane 1

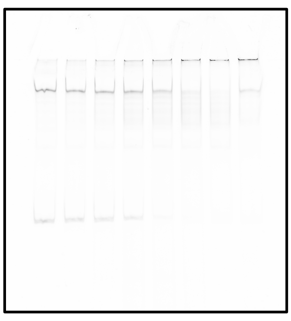

Lane 1,4,7 belong to EDF2d lane 2,3,4

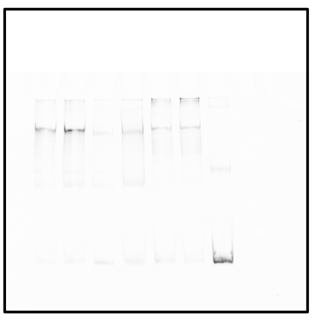

Image belongs to EDF2e-FAM

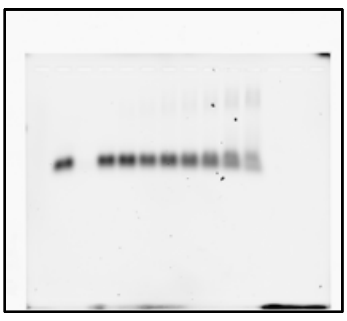

Image belongs to EDF2e-Cy3

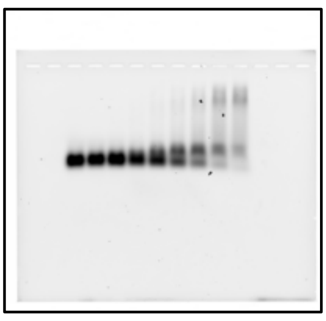

Lane 1-8 belong to EDF2g - red, lane 1-8

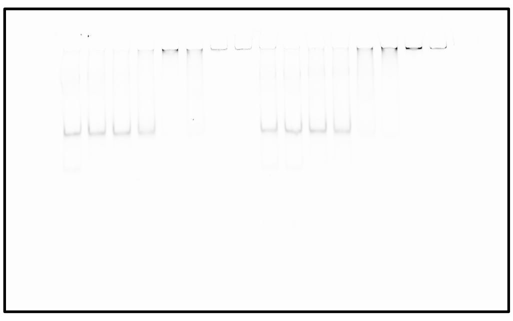

Lane 1-8 belong to EDF2g - green, lane 1-8

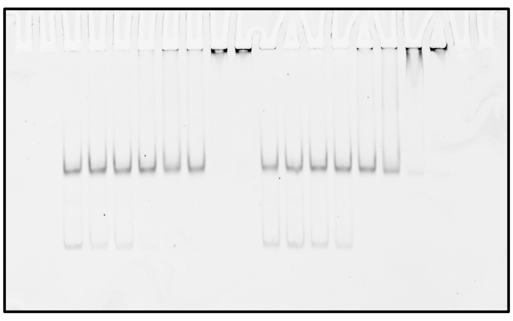

Lane 1-20 belong to EDF2hi,i lane 1-20

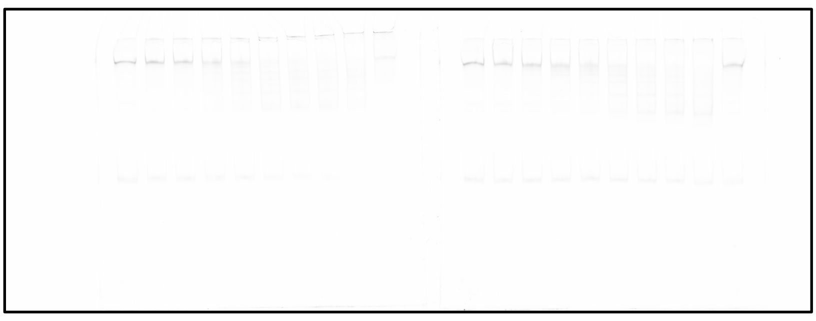

Lane 1-6 belong to EDF2j upper panel lane 1-6

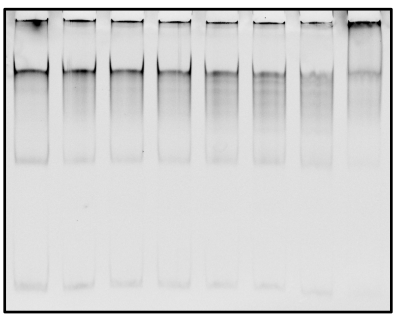

Lane 1-6 belong to EDF2j lower panel lane 1-6

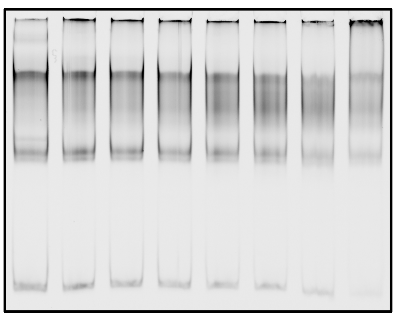

Lane 5-10 belong to EDF2k lane 1-6

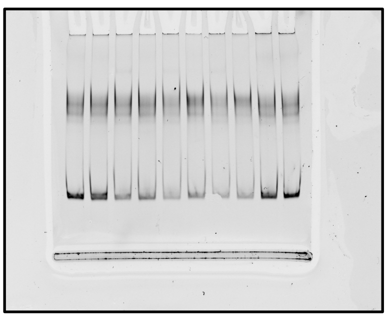

Lane 3-6,9,10 belong to EDF2l lane 1-6

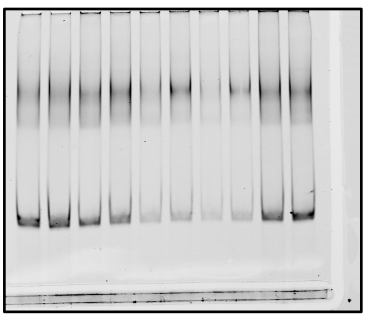

EDF 3:

Lane 1-4belong to EDF3a lane 1-4

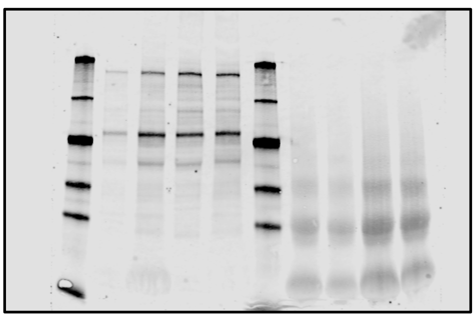

Image belongs to EDF3c upper panel

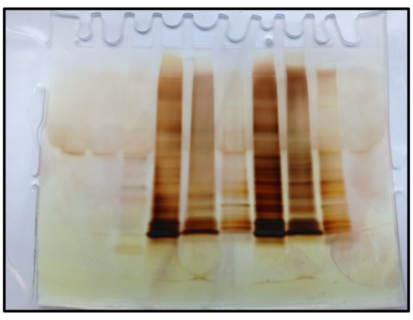

Image belongs to EDF3c lower panel

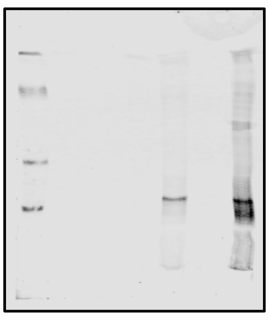

Image belongs to EDF3f

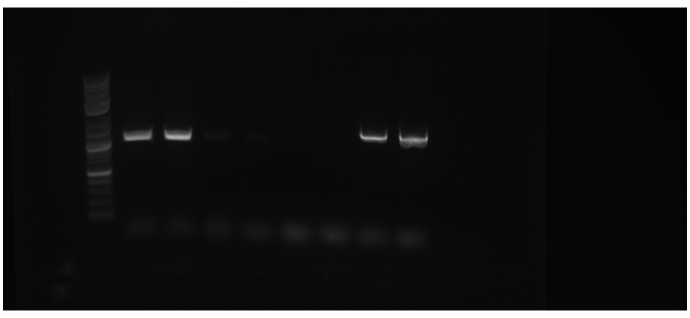

Image belongs to EDF3h upper panel

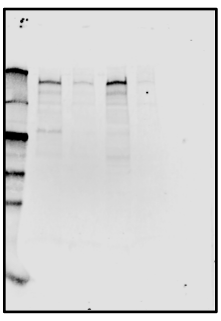

Image belongs to EDF3h lower panel

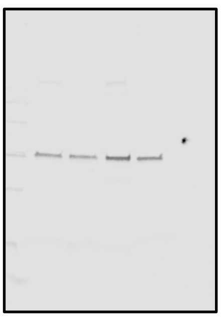

EDF 10:

Images belong to EDF10c

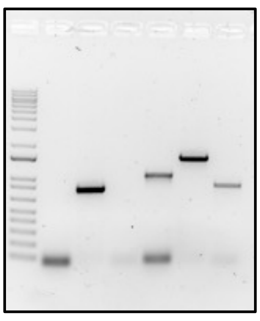

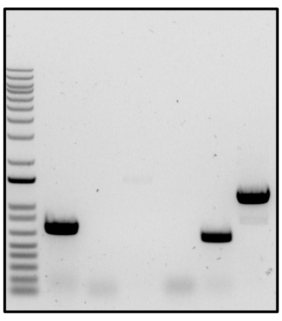

Supplement: Supplementary file 1 — This file contains Supplementary Notes 1 and 2, Supplementary References and Supplementary Fig. 1. [file 41586_2025_8595_MOESM1_ESM.pdf]
